# Downregulation of TNF-*α*/TNF-R1 Signals by AT-Lipoxin A4 May Be a Significant Mechanism of Attenuation in SAP-Associated Lung Injury

**DOI:** 10.1155/2019/9019404

**Published:** 2019-04-11

**Authors:** Suhui Yu, Jianming Xie, Yukai Xiang, Shengjie Dai, Dinglai Yu, Hongwei Sun, Bicheng Chen, Mengtao Zhou

**Affiliations:** ^1^Department of Surgery, The First Affiliated Hospital of Wenzhou Medical University, Wenzhou, Zhejiang Province, China; ^2^Department of Surgery, Ningbo First Hospital, Ningbo, Zhejiang Province, China; ^3^Key Laboratory of Diagnosis and Treatment of Severe Hepato-Pancreatic Diseases of Zhejiang Province, Zhejiang Provincial Top Key Discipline in Surgery, Wenzhou, Zhejiang Province, China

## Abstract

Our previous studies verified the potent anti-inflammatory effects against severe acute pancreatitis (SAP) of AT-Lipoxin A4 and their analogues. However, the anti-inflammatory effects of AT-Lipoxin A4 on SAP-associated lung injury are not thoroughly known. We used western blot, polymerase chain reaction (PCR), and immunofluorescence to investigate the downregulation of TNF-*α* signals in cellular and animal models of SAP-associated lung injury following AT-Lipoxin A4 intervention. *In vitro*, we found that AT-Lipoxin A4 markedly suppressed protein expression in TNF-*α* signals in human pulmonary microvascular endothelial cell, such as tumor necrosis factor receptor-associated factor 2 (TRAF2), TNF-R1-associated death domain (TRADD), receptor-interacting protein (RIP), vascular cell adhesion molecule-1 (VCAM-1), and E-selectin. Moreover, AT-Lipoxin A4 inhibited downstream signals activated by TNF-*α*, including NF-*κ*B/p65, JNK/MAPK, and ERK/MAPK. *In vivo*, AT-Lipoxin A4 significantly decreased pathological scores of the pancreas and lungs and the serum levels of IL-6 and TNF-*α*. Immunofluorescence, western blotting, and real-time PCR assay showed that AT-Lipoxin A4 significantly attenuated the expression of TNF-R1, TRADD, TRAF2, and RIP in the lungs of SAP rats. In addition, the activation of NF-*κ*B was also downregulated by AT-Lipoxin A4 administration as compared with SAP rats. AT-Lipoxin A4 could inhibit the production of proinflammatory mediators and activation of TNF-*α* downstream signals such as NF-*κ*B and MAPK. Downregulation of TNF-*α* signals by AT-Lipoxin A4 may be a significant mechanism in the attenuation of SAP-associated lung injury.

## 1. Introduction

A challenge exists in the early clinical treatment of severe acute pancreatitis (SAP) in terms of how to control the occurrence of pancreatitis-induced multiple organ dysfunction syndrome (MODS), as a common end-stage pathophysiological process of SAP and a major cause of early death of patients with SAP [1]. MODS often begins with acute lung injury (ALI) and acute respiratory distress syndrome (ARDS). Recently, there has been an increasing awareness that systemic inflammatory response syndrome (SIRS) plays a key role in SAP-induced ALI [2]. Nevertheless, the clinical occurrence of ALI in SAP is still unpredictable [3]. So SIRS and subsequent lung injury as important experimental topics have not been totally resolved.

As a barrier between blood circulation and organization, the vascular endothelium plays a pivotal role in the occurrence and development of inflammation. Pulmonary microvascular inflammation, which involves the production of cytokines and upregulation of adhesion molecules on endothelial cells, is one of the early stage manifestations during the pathogenesis of ALI [4]. Studies have shown that SAP-induced SIRS and excessive leakage of pulmonary polymorphonuclear neutrophils (PMN) are the key steps of the occurrence of lung injury [5, 6]. In this study, we further explored how to effectively inhibit the excessive inflammatory response in vascular endothelial cells and also investigated the underlying pathophysiological mechanisms.

TNF-*α* is generally derived from activated fixed-tissue macrophages and circulating monocytes. There are various kinds of inflammatory factors, among which TNF-*α* is discovered earlier and playing an important part in the development of SAP and the occurrence of systemic complications [7]. In the inflammatory cascade, TNF-*α* is thus considered a proximal mediator that regulates the synthesis of several cytokines, chemokines, and adhesion molecules in endothelial cells and neutrophils [5]. TNF-*α* plays a central role in SIRS and is associated with lung injury caused by acute necrotizing pancreatitis. The TNF-*α* pathway is divided into apoptosis pathways and proinflammatory pathways, including the TNF-R2 (P75) and TNF-R1 (P55) paths [8]. TNF-*α* works by binding to TNF-R1, subsequently docked by adaptor proteins such as TNFR1-associated death domain protein (TRADD), TNFR-associated factor 2 (TRAF2), and receptor-interacting protein (RIP) and then triggers a series of intracellular events that ultimately results in the activation of a major transcription factor, nuclear factor *κ*B (NF-*κ*B) [9, 10]. The activation of NF-*κ*B can in turn enhance the transcription of the TNF-*α* gene, thereby forming a vicious feedback loop that is able to amplify the early inflammatory signals and aggravate the initial inflammatory effects [11].

Lipoxins (LXs) are synthesized locally from arachidonic acid at an inflammatory site by transcellular biosynthesis and considered to act as an “inflammatory braking signal” in inflammation by limiting the trafficking of leukocytes to the inflammatory site [12, 13]. Lipoxin A4 has diverse actions on PMNs by attenuating their chemotaxis, adhesion, and transmigration across vascular endothelial cells and epithelial cells [14]. Additionally, it has also been reported that LXA4 ameliorated pathological changes in both the pancreas and lungs in SAP [15]. Furthermore, our previous study confirmed that LXA4 could inhibit the production of proinflammatory mediators in human pulmonary microvascular cells (HPMECs) exposed to TNF-*α*. The anti-inflammatory effects of LXA4 may be dependent on the inhibition of NF-*κ*B/p65 and p38/MAPK pathways, both of which are downstream signaling of TNF-*α* and its receptor 1 pathway. Using of LXA4 to inhibit inflammation and protect the target organ is a new strategy for the treatment of acute necrotizing pancreatitis, although the specific mechanism is not fully elucidated.

As a more stable analog of LXA4, AT-Lipoxin A4 shares many anti-inflammatory activities with the native LXA4 [16]. However, the potential mechanisms of AT-Lipoxin A4 in attenuating SAP-associated lung injury remain unknown. In this study, we investigated the role of AT-Lipoxin A4 that played on protecting against pancreatitis-induced lung injury and clarified whether this effect is through interfering with the TNF-*α*/TNF-R1 pathway.

## 2. Materials and Methods

### 2.1. Cell Culture

HPMECs were purchased from American Type Culture Collection (ATCC) and maintained in PRMI-1640 (Gibco, NY, USA) supplemented with 10% FBS (Gibco), 100 U/mL penicillin, and 100 *μ*g/mL streptomycin (Solarbio, Beijing, China) at 37°C in a humidified atmosphere with 5% CO_2_. For the experiments, HPMECs were seeded onto 96-well plates, 6-well plates, or 60 mm culture dishes.

### 2.2. Lentiviral Packaging and Transfection

In this study, a lentiviral vector was constructed to specifically silence TNF-*α*/TNF-R2 (p75) expression in the HPMECs. Two groups were set up for the cell transfection: the control group without the transfection and the TNF-*α*/TNF-R2 group with lentivirus target sequence interference. The p75-specific shRNA-encoding pLenti6.3-GFP vector, pGag-pol vector, pRev vector, and Pvsvg vector (R&S Biotechnology, Shanghai, China) were cotransfected into the 293T cell line using ViraPower™ Lentiviral Expression Systems (Invitrogen, Carlsbad, CA) to produce lentivirus stocks with a negative construct as a negative control. The 293T cells were harvested after transfection for 48 hours, and the cell debris was removed by centrifugation at 4°C. The crude viral extract was filtered, and centrifuge enrichment was conducted.

### 2.3. Model Preparation and Specimen Collection

A total of 48 healthy male Sprague-Dawley (SD) rats (weighing 250 ± 20 g, 6–8 weeks old) were provided by the Laboratory Animal Center of Wenzhou Medical College. The rats were bred and housed in standard shoe-box cages in a climate-controlled room with an ambient temperature of 20–22°C and a 12 h light-dark cycle. They were treated in accordance with the protocols approved by the Institutional Animal Committee of Wenzhou Medical College. All animals received care in accordance with the Guide for the Care and Use of Laboratory Animals.

The SAP rat model was established according to Zhao et al. [17]. Briefly, severe acute pancreatitis was induced by the intraductal infusion of 5% sodium taurocholate (0.1 mL/100 g), by using an infusion pump at a speed of 0.1 mL/min, following clamping of the proximal end of the common bile duct and cannulating the biliary-pancreatic duct by a thin polyethylene catheter. The sham rats the sodium taurocholate injection were omitted, but the surgical procedure was identical to the other groups, including bile duct cannulation. The 48 experimental animals were randomly divided into three groups, including the sham group (*N* = 16), the SAP group (*N* = 16), and the AT-Lipoxin A4 group (*N* = 16). Rats in the AT-Lipoxin A4 (Cayman, USA) group were injected with 0.1 mg/kg of AT-Lipoxin A4 (dissolved in saline) through the femoral vein 30 minutes following the procedure, while those in the other two groups were give normal saline in the same manner. In order to reduce the influence of alcohol on the experimental results, we used a self-made low-flow liquid nitrogen device to wash AT-Lipoxin A4 (Cayman, USA) in a sterile experimental table. AT-Lipoxin A4 (100 *μ*g) in normal saline (1 mL) was dissolved in an EP tube and rinsed with liquid nitrogen rinsing for about 3 minutes. Eight rats from each group were randomly submitted for sacrifice after 12 and 24 h following the induction of SAP.

The surgical procedure for sham rats was identical to those for the other groups, including bile duct cannulation. The blood of the inferior vena cava was collected and centrifuged at 4°C, and the serum was stored at -80°C for further studies. Fresh pancreatic and lung tissues were removed, fixed with 4% formaldehyde or frozen in liquid nitrogen overnight, and then stored at -80°C.

### 2.4. Histopathologic Analysis

Formaldehyde-fixed pancreatic and pulmonary tissue blocks were embedded in paraffin, sectioned and stained with hematoxylin and eosin (H&E). All microscopic sections were examined by two independent pathologists who were blinded to the experimental protocol. Pancreatitis severity was scored according to a protocol described by Schmidt et al. [18]. The pathological changes in the lungs were scored according to a protocol described by Mayer et al. [19]. Briefly, inflammatory cell infiltrate and architectural damage were graded from 0 to 3 (0; normal, 3: severe).

### 2.5. Enzyme-Linked Immunosorbent Assay (ELISA)

Rat serum cytokines including interleukin-6 (IL-6) and TNF-*α* were determined by ELISA using kits (Affymetrix, USA) and performed following the instructions provided by the manufacturer. Concentrations of IL-6 and TNF-*α* were expressed in picograms per milliliter of the serum.

### 2.6. RNA Isolation and Quantitative Real-Time PCR

Total RNA was isolated from tissue samples using a TRIzol Kit and was subjected to reverse transcription using oligo-dT-primed RT following the manufacturer's instructions. Quantitative real-time PCR was performed using a SYBR Green real-time PCR Master Mix (Toyobo, Japan) on a 7500 Real-Time PCR System (Applied Biosystems, USA). Primer sequences are listed in Table 1.

### 2.7. Protein Extraction and Western Blot Analysis

For whole cell extraction, cells and tissue samples were lysed in radioimmune precipitation assay buffer (Beyotime, Beijing, China) supplemented with protease and phosphatase inhibitor. Equal amounts of proteins were separated by 10% SDS-PAGE and transferred to a polyvinylidene difluoride membrane. After being blocked with 5% nonfat dry milk, membranes were incubated overnight at 4°C with primary antibodies against VCAM-1, E-selectin, ERK, P-ERK, JNK1/2, p-JNK1/2, TNF-R1, TRAF2, NF-*κ*B/P65, RIP, GAPDH, and beta-actin that were diluted at the indicated concentration. Then, the membranes were washed three times with TBST (10 mmol/L Tris-HCl, pH 8.0, containing 150 mmol/L NaCl and 0.1% Tween-20) and incubated with secondary antibodies (Merck Millipore, Darmstadt, Germany) for 1.5 h at room temperature. After washing for 3 × 10 min with TBST, target bands were developed using ECL (Advansta, California, USA) and exposed on a film (Kodak, NY, USA). The intensity of the protein bands was measured using Quantity One version 4.6.2 Image software (Bio-Rad, USA).

The anti-GAPDH primary antibody was purchased from Bioworld (St. Louis Park, MN, USA). The anti-p-JNK1/2, TRAF2, NF-*κ*B/P65, and beta-actin primary antibodies were purchased from Abcam (MA, USA). All other primary antibodies were purchased from Cell Signaling Technology (Danvers, MA, USA).

### 2.8. Immunofluorescence

Frozen tissue sections of 8 *μ*m thickness were fixed in 4% paraformaldehyde for 15 minutes and then rinsed with Tris-buffered saline (TBS). After being blocked with 5% goat serum in TBS for 1 h at 37°C, the sections were then incubated with primary antibodies of NF-*κ*B and incubated overnight at 4°C. After washing thrice with TBS, the slides were then incubated with a secondary antibody for 1 h at 37°C. Sections were then washed with TBS and incubated with the DAPI to highlight cell nuclei. Slides were visualized under a fluorescent microscope (Leica Microsystems, Germany).

### 2.9. Statistical Analysis

Data are expressed as the mean ± SEM. Differences between mean values were analyzed by one-way analysis of variance. Dunnett's post hoc test was used for multiple group comparisons. *P* < 0.05 was considered statistically significant.

## 3. Results

### 3.1. Silence of TNF-R2 by Lentivirus Transfection in the HPMECs

**Figure 1:**
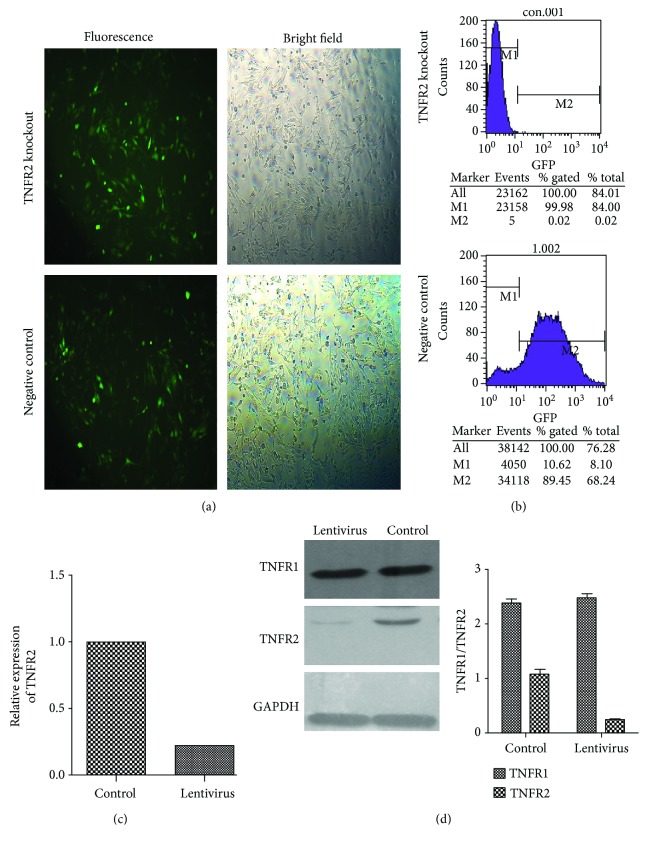
Silence of TNF-R2 by lentivirus transfection in the HPMECs. 20 *μ*L/well of lentivirus with a titre of 2.5^∗^109 TU/mL was added to the HPMEC suspension in a 6-well cell culture plate and cultured for 24 h. (a) The expression of the GFP lentivirus reporter gene was observed by fluorescence microscopy, and cellular morphology was observed with bright field microscopy. (b, c, d) The transfection rate was determined by flow cytometry, PCR, and Western blot. Flow cytometry determination of lentivirus transfection efficiency was 90%. PCR showed interference with an efficiency of 78%, and Western blot results showed interference with an efficiency of 77%.

Flow cytometry determination of lentivirus transfection efficiency was 90% (Figure 1(b)). PCR results showed interference with an efficiency of 78% (Figure 1(c)), and Western blot results showed interference with an efficiency of 77% (Figure 1(d)) and thus can be used for subsequent experiments.

### 3.2. Downregulation of RIP and TRAF2 Expression in TNF-*α*-Stimulated TNF-R2^(-/-)^ HPMECs by AT-Lipoxin A4

**Figure 2:**
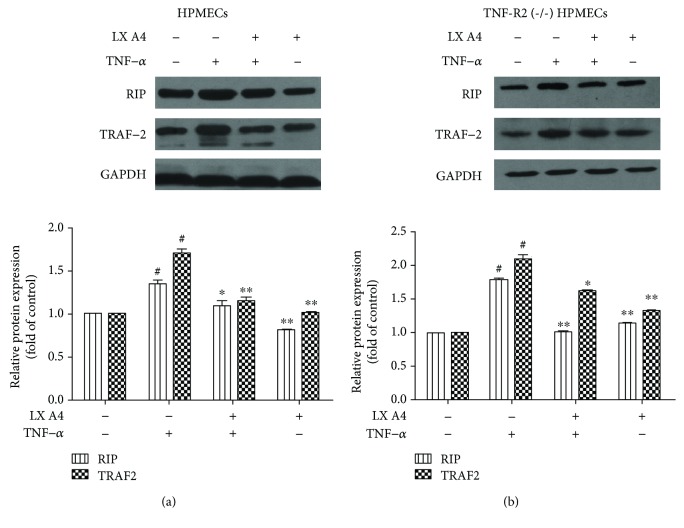
AT-Lipoxin A4 downregulation of RIP and TRAF2 expression in TNF-*α*-stimulated HPMECs and TNF-R2^(-/-)^ HPMECs. HPMECs and TNF-R2^(-/-)^ HPMECs were pretreated with AT-Lipoxin A4 (50 ng/mL) for 1 h followed by incubation with TNF-*α* (50 ng/mL) for 6 h. (a) Western blot assay shows expression of RIP and TRAF2 in HPMECs. (b) Western blot assay shows expressions of RIP and TRAF2 in TNF-R2^(-/-)^ HPMECs. GAPDH was used as an internal control. The data showed representatives of at least three independent experiments. ^#^*P* < 0.01 compared with the control, ^∗^*P* < 0.05 compared with TNF-*α* in the absence of AT-Lipoxin A4, and ^∗∗^*P* < 0.01 compared with TNF-*α* in the absence of AT-Lipoxin A4.

The cytotoxicity experiments of AT-Lipoxin A4 were performed at 0-70 ng/mL, and the cell viability test showed that AT-Lipoxin A4 is nontoxic to HPMECs at the dosages used in this study (data not shown). Before each experiment, cells were grown in RPMI-1640 supplemented with 1% FBS for 12 h. Then, cells were treated with 50 ng/mL AT-Lipoxin A4 or vehicle (0.035% ethanol) for 1 h before the addition of 50 ng/mL TNF-*α*. The effect of TNF-*α* on JNK/MAPK and ERK/MAPK phosphorylation was assessed at 15 min. Besides, cells were preincubated with or without AT-Lipoxin A4 for 1 h before stimulation with TNF-*α* for various periods: 15 min for the measurement of NF-*κ*B/p65 phosphorylation; 1 h for RIP, TRAF2, and TRADD translocation; and 6 h for the measurement of protein expression of VCAM-1, E-selectin, RIP, and TRAF2. To investigate whether the inhibition of inflammation by AT-Lipoxin A4 was involved in the regulation of RIP and TRAF2, we examined the expressions of RIP and TRAF2 of HPMECs using western blot assay. We found that the expression of RIP and TRAF2 was increased significantly following TNF-*α* stimulation compared with the control (*P* < 0.01) in HPMECs, whereas AT-Lipoxin A4 downregulated RIP and TRAF2 expression in TNF-*α*-stimulated HPMECs (Figure 2(a)). In addition, HPMECs infected with the indicated lentivirus (TNFR2^(-/-)^ HPMECs) were pretreated with AT-Lipoxin A4 (50 ng/mL) for 1 h and then stimulated with TNF-*α* (50 ng/mL). The expression of RIP and TRAF2 was examined by western blot assay. And we found AT-Lipoxin A4 downregulated RIP and TRAF2 expression in TNF-*α*-stimulated TNFR2^(-/-)^ HPMECs (Figure 2(b)).

### 3.3. Inhibition of TNF-*α*-Induced Activation of JNK/MAPK and ERK/MAPK by AT-Lipoxin A4

**Figure 3:**
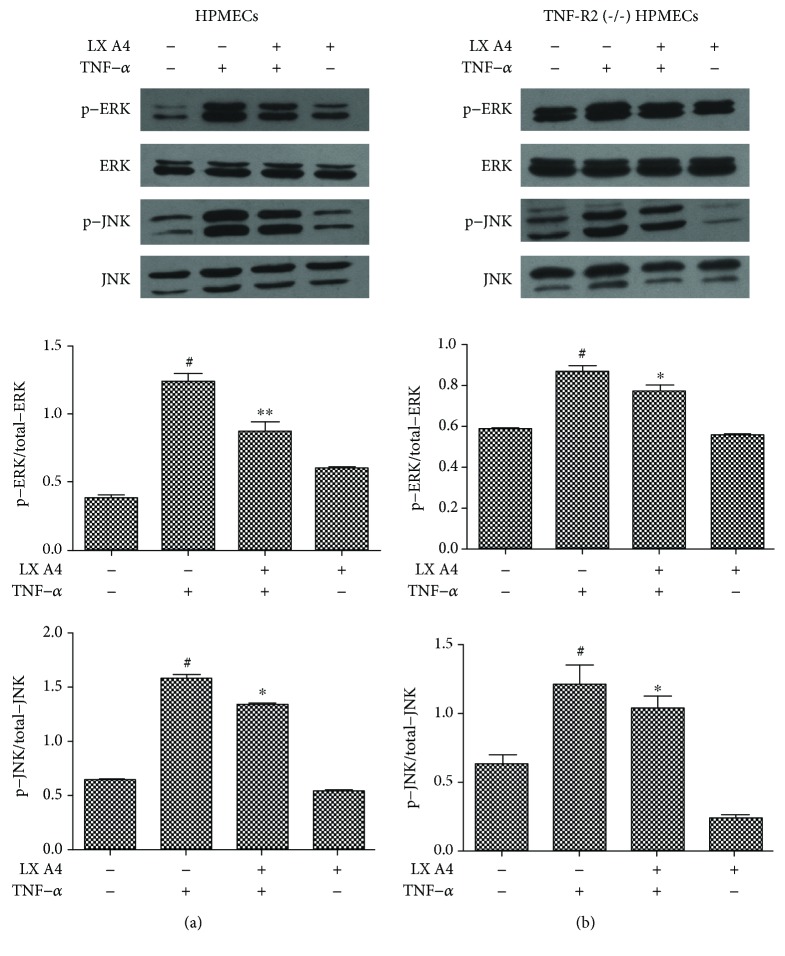
Inhibition of TNF-*α* induced activation of JNK/MAPK and ERK/MAPK by AT-Lipoxin A4. HPMECs and TNF-R2^(-/-)^ HPMECs were pretreated with AT-Lipoxin A4 (50 ng/mL) for 1 h and then stimulated with TNF-*α* (50 ng/mL) for another 15 min. (a) Western blot assay shows expressions of p-ERK, ERK, p-JNK, and JNK in HPMECs. (b) Western blot assay shows expressions of p-ERK, ERK, p-JNK, and JNK in TNF-R2^(-/-)^ HPMECs. The data showed representatives of at least three independent experiments. ^#^*P* < 0.01 compared with the control, ^∗^*P* < 0.05 compared with TNF-*α* in the absence of AT-Lipoxin A4, and ^∗∗^*P* < 0.01 compared with TNF-*α* in the absence of AT-Lipoxin A4.

Previous studies have shown that AT-Lipoxin A4 inhibition of the TNF-*α* induced p38 MAPK activation [20]. To investigate whether the inhibition of inflammation by AT-Lipoxin A4 was involved in the regulation of JNK/MAPK and ERK/MAPK pathways, we examined the phosphorylation of JNK/MAPK and ERK/MAPK after stimulation of TNF-*α* in HPMECs using western blot assay. A 15 min treatment of TNF-*α* was determined as previous studies have shown that TNF-*α* causes MAPK phosphorylation within 15 min in human endothelial cells [11, 12]. We found that TNF-*α* induced a marked increase in ERK and JNK phosphorylation in HPMECs and these effects of TNF-*α* were inhibited by AT-Lipoxin A4 in HPMECs (Figure 3(a)). And we examined the phosphorylation of JNK/MAPK and ERK/MAPK in TNF-*α*-induced TNFR2^(-/-)^ HPMECs using western blot analysis. We found that TNF-*α* induced a marked increase in JNK and ERK phosphorylation in TNFR2^(-/-)^ HPMECs. AT-Lipoxin A4 reduced TNF-*α*-induced phosphorylation of JNK and ERK in TNFR2^(-/-)^ HPMECs (Figure 3(b)).

### 3.4. Downregulation of VCAM-1 and E-selectin Expression in TNF-*α*-Stimulated HPMECs by AT-Lipoxin A4

**Figure 4:**
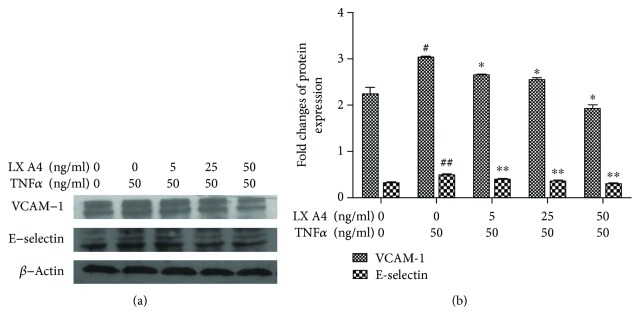
Downregulation of VCAM-1 and E-selectin expression in TNF-*α*-stimulated HPMECs by AT-Lipoxin A4. HPMECs were pretreated with AT-Lipoxin A4 (5, 25, and 50 ng/mL) for 1 h followed by incubation with TNF-*α* (50 ng/mL) for 6 h. (a, b) Western blot assay shows expressions of VCAM and E-selectin in HPMECs. The data showed representatives of at least three independent experiments. ^#^*P* < 0.01 compared with the control, ^##^*P* < 0.05 compared with the control, ^∗^*P* < 0.05 compared with TNF-*α* in the absence of AT-Lipoxin A4, and ^∗∗^*P* < 0.01 compared with TNF-*α* in the absence of AT-Lipoxin A4.

**Figure 5:**
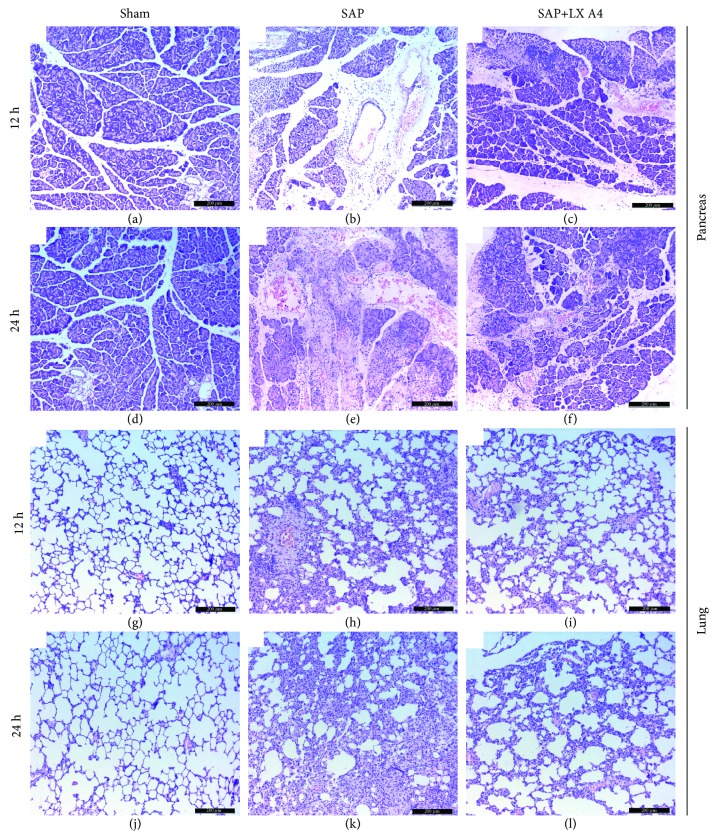
Attenuation of histopathological injury of the pancreas and lung in SAP model by AT-Lipoxin A4 administration. (a–f) H&E staining shows changes of the pancreas at 12 h or 24 h after the procedure. (g–l) H&E staining shows changes of the lungs at 12 h or 24 h after the procedure.

Our previous studies found that AT-Lipoxin A4 downregulation of ICAM-1 expression in TNF-a stimulated HPMECs [20]. The effects of AT-Lipoxin A4 on TNF-*α*-induced expression of VCAM-1 and E-selectin on the surface of the HPMECs were evaluated by western blot assay. Exposure of cells to TNF-*α* for 6 h induced significantly increased expressions of VCAM-1 and E-selectin. Pretreatment with AT-Lipoxin A4 significantly inhibited TNF-*α*-induced expressions of VCAM-1 and E-selectin in a dose-dependent manner (Figures 4(a) and 4(b)). Attenuation of histopathological injury of the pancreas and lung in the SAP model by AT-Lipoxin A4 administration. Both at 12 h and 24 h after SAP occurred, obvious increases in inflammatory cell infiltration, edema, acinar cell vacuolization, and acinar cell necrosis were observed in the pancreatic tissues in the SAP rats by H&E staining, compared with the sham rats (Figures 5(a), 5(b), 5(d), and 5(e)). The pathological changes in the AT-Lipoxin A4 group were relieved compared with those in the SAP group (Figures 5(b), 5(c), 5(e), and 5(f)). The Schmidt standard was applied to score the pancreatic injury of experimental rats. At 12 h, the injury score in the sham, SAP, and AT-Lipoxin A4 groups were 0.42 ± 0.09, 11.34 ± 1.23, and 9.73 ± 1.08, respectively, while the pathological scores were 0.46 ± 1.07, 13.38 ± 1.34, 10.23 ± 1.42 at 24 h, respectively (Table 2). Histopathological injury of the pancreas in the SAP model is significantly attenuated by AT-Lipoxin A4 (*P* < 0.05). Histopathological changes in lung tissues were scored as described by Mayer et al. [19]. Significant alveolar thickening, vasocongestion, and leukocyte infiltration were observed in the SAP group compared with the control group (Figures 5(g), 5(h), 5(j), and 5(k)), while these were attenuated by AT-Lipoxin A4 administration (Figures 5(i) and 5(l)). According to the lung injury score, AT-Lipoxin A4 significantly reduced scores as compared to the SAP group (Table 2), and we confirmed that AT-Lipoxin A4 significantly alleviated histopathologic changes of SAP-induced lung injury.

### 3.5. Downregulated TNF-*α*/TNF-R1 Signal in Lung Tissues by AT-Lipoxin A4 Administration

**Figure 6:**
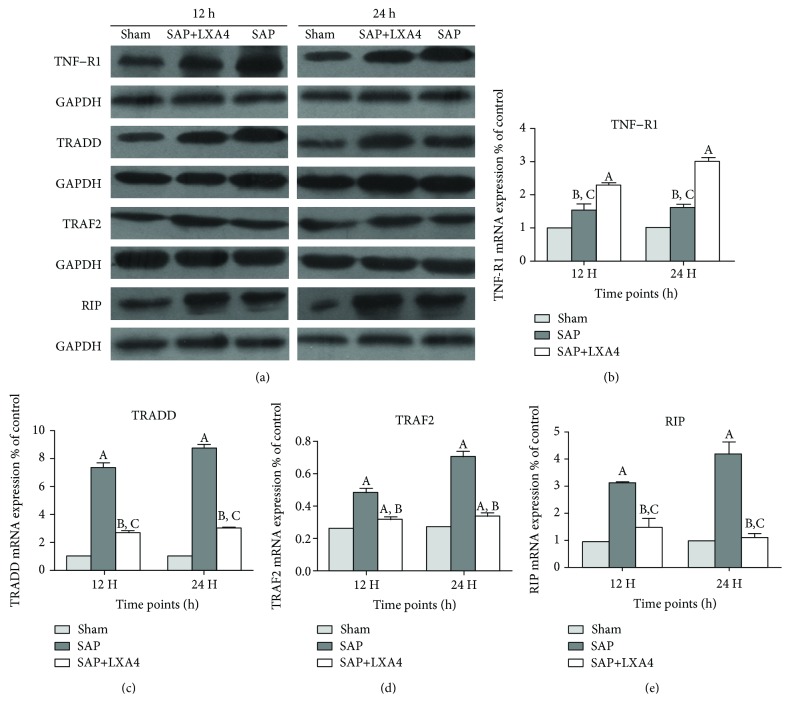
Downregulation of TNF-*α*/TNF-R1 signal in lung tissues by AT-Lipoxin A4 administration. (a–e) Western blot assay shows expressions of total TNF-R1, TRADD, TRAF2, and RIP in lung tissue at 12 h or 24 h after the procedure. GAPDH was used as an internal control. Data represent the mean ± SEM (*n* = 8). (A) *P* < 0.05, SAP vs. sham; (B) *P* < 0.05, SAP+AT-Lipoxin A4 vs. sham; (C) *P* < 0.05 SAP+AT-Lipoxin A4 vs. SAP.

It is well known that TNF-*α* exerts its biological functions via interaction with its' cognate membrane receptors, namely, TNF-R1 and TNF-R2. TNF-*α* binds to TNF-R1, subsequently docked by adaptor proteins TRADD, TRAF2, and RIP and then triggers a series of intracellular events that ultimately activates proinflammatory signal transduction pathways [21]. Therefore, we performed western blot assays to determine the possible roles of TNF-R1, TRADD, TRAF2, and RIP played in this process. The sham group did present a low expressional level of TNF-R1, TRADD, TRAF2, and RIP, whereas strong intensity expression of those proteins were consistently observed in the SAP group and AT-Lipoxin A4 group in lung tissues at two time points. However, treatment with AT-Lipoxin A4 caused a significant downregulation of the expression of those signaling molecules as compared to the SAP group (Figure 6).

### 3.6. Inhibition of NF-*κ*B/p65 Activation in TNF-R2^(-/-)^ HPMECs and Lung Tissue by AT-Lipoxin A4

**Figure 7:**
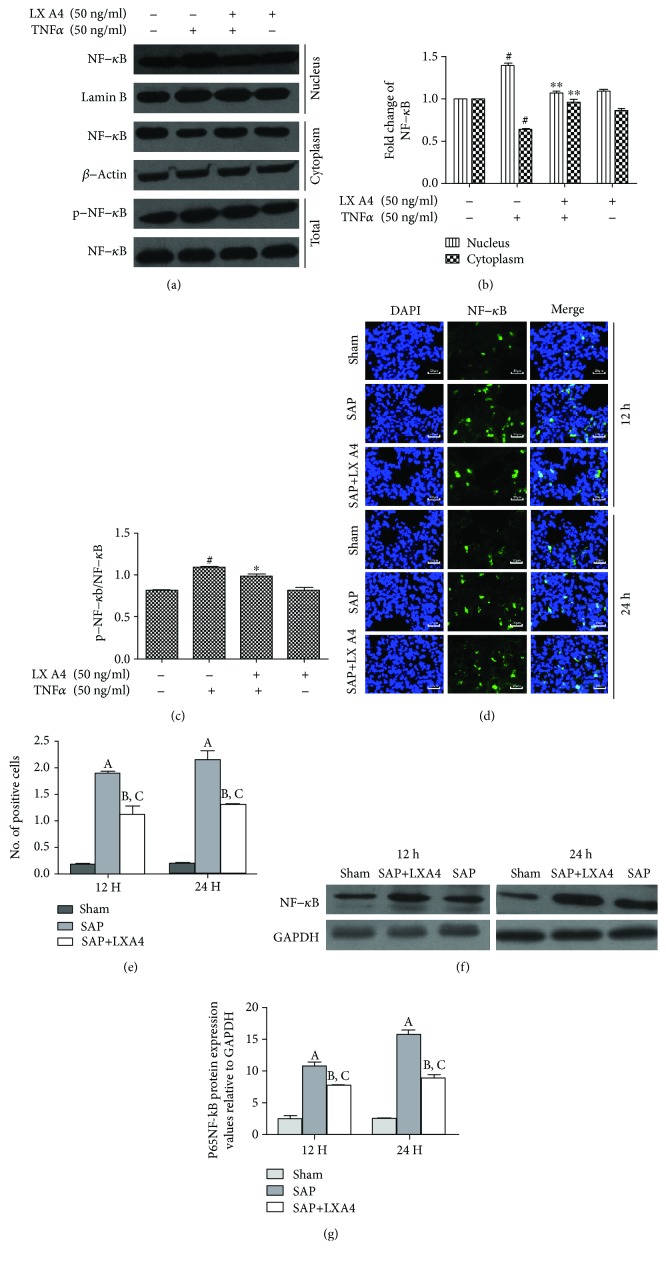
Inhibition of NF-*κ*B/p65 activation in TNF-R2^(-/-)^ HPMECs and lung tissue by AT-Lipoxin A4. TNF-R2^(-/-)^ HPMECs were pretreated with AT-Lipoxin A4 (50 ng/mL) for 1 h and then stimulated with TNF-*α* (50 ng/mL) for 15 min. (a–c) Western blot assay shows expressions of nuclear NF-*κ*B, cytoplasmic NF-*κ*B, and total p-NF-*κ*B in TNF-R2^(-/-)^ HPMECs. (d, e) Single and merged images show immunofluorescence staining of NF-*κ*B (green) in lung tissue at 12 h or 24 h after the procedure. The cell nucleus is stained blue by DAPI. (f, g) Western blot assay shows expressions of total NF-*κ*B in lung tissue at 12 h or 24 h after the procedure. Beta-actin and Lamin B were used as internal controls, respectively. The data showed representatives of at least three independent experiments. ^#^*P* < 0.01 compared with the control, ^∗∗^*P* < 0.01 compared with TNF-*α* in the absence of AT-Lipoxin A4, and ^∗^*P* < 0.05 compared with TNF-*α* in the absence of AT-Lipoxin A4.

NF-*κ*B is a transcriptional factor that is essential for the gene expression of inflammatory mediators in AP-associated ALI. Our previous study showed that LXA4 suppressed TNF-*α*-induced phosphorylation and translocation of NF-*κ*B/p65 in HPMECs. In this experiment, we found that AT-Lipoxin A4 markedly reduced the NF-*κ*B/p65 level in both the nucleus and cytoplasm in TNF-*α*-stimulated TNF-R2^(-/-)^ HPMECs (Figures 7(a) and 7(b)). In addition, western blot showed that the phosphorylation of NF-*κ*B/p65 was increased significantly following TNF-*α* stimulation compared with the control in TNF-R2^(-/-)^ HPMECs, while this effect was significantly inhibited by AT-Lipoxin A4 (Figures 7(a) and 7(c)). Thus, AT-Lipoxin A4 inhibited TNF-*α*-induced NF-*κ*B/p65 activation in TNFR-2^(-/-)^ HPMECs.

Via immunofluorescence marking, we found that SAP-associated lung injury caused the nuclear translocation of NF-*κ*B at 12 h and 24 h. However, treatment with AT-Lipoxin A4 significantly inhibited NF-*κ*B nuclear translocation at both time points (Figures 7(d) and 7(e)). Moreover, the presence of activated NF-*κ*B in the nucleus was further confirmed by western blot. Only low levels of NF-*κ*B were found in the sham group at 12 h and 24 h. As expected, in the SAP group, the overexpression of the NF-*κ*B was detected (*P* < 0.01, 12 h; *P* < 0.01, 24 h). AT-Lipoxin A4 treatment resulted in a significant reduction of NF-*κ*B compared to the SAP group (*P* < 0.05, 12 h; *P* < 0.05, 24 h) (Figures 7(f) and 7(g)).

### 3.7. Downregulated Levels of Proinflammatory Cytokines in SAP by AT-Lipoxin A4 Administration

**Figure 8:**
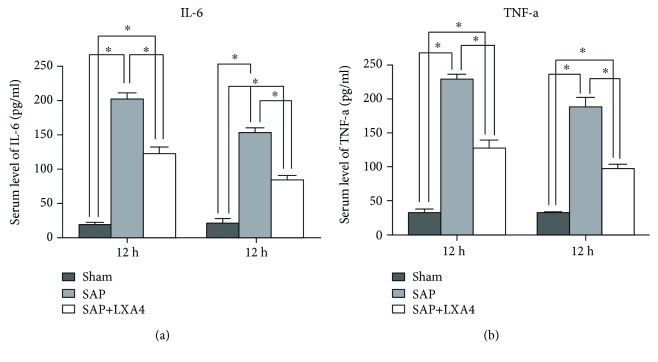
Downregulated levels of proinflammatory cytokines in SAP by AT-Lipoxin A4 administration. (a) Serum IL-6 was increased significantly in the SAP group compared with the sham group. AT-Lipoxin A4 treatment significantly reduced the levels of serum IL-6. (b) Serum TNF-*α* was increased significantly in the SAP group. AT-Lipoxin A4 treatment significantly reduced the levels of serum TNF-*α*. ^∗^*P* < 0.05.

Inflammatory mediators, such as TNF-*α* and IL-6, have been shown to significantly increase during SAP [22, 23], which determines typical systemic effects such as ARDS in SAP [24]. Therefore, we measured the levels of TNF-*α* and IL-6 in serum. In the sham group, very low or undetectable levels of TNF-*α* and IL-6 were determined at both time points. As expected, significant high levels of TNF-*α* and IL-6 were present in SAP, and the higher level appeared at 12 h and then declined at 24 h. The AT-Lipoxin A4 group had the same pattern of changes as the SAP group, but the levels of both proinflammatory mediators were profoundly downregulated as compared to those of the SAP group (Figure 8).

## 4. Discussion

In our previous study, we provided evidence that AT-Lipoxin A4 ameliorates SAP and SAP-associated lung injury. AT-Lipoxin A4 can alleviate pathological changes in the pancreas and lungs in SAP, as well as decrease the levels of serum proinflammatory mediators, ICAM-1 expression, and activation of NF-*κ*B [15]. However, the mechanism for how LXA4 attenuates SAP-associated lung injury is still unknown. In the present study, we found that AT-Lipoxin A4 significantly suppressed the expression of TNF-R1 signaling-associated proteins, like TRADD, TRAF2, and RIP, and inhibited downstream of TNF-*α*/TNFR1 signaling, like NF-*κ*B, JNK/MAPK, and ERK/MAPK in lung tissues and HPMECs. Taken together, the outcomes of the present study indicate that AT-Lipoxin A4 provides protection against SAP-associated lung injury by inhibiting activation of TNF-*α* signals.

TNFR1 signaling depends on the ligand-induced formation of intracellular signaling complexes consisting of TRADD, TRAF2, RIP, and FADD, each exerting a different and specific function to activate the signaling [9, 25, 26]. In our research, we found that the expression of TNF-R1, TRADD, TRAF2, and RIP in lung tissues was increased in SAP rats while AT-Lipoxin A4 suppressed their expression. *In vitro*, we found that AT-Lipoxin A4 also markedly suppressed the protein expression of TRAF2 and RIP in TNF-*α*-exposed HPMECs and TNF-R2^(-/-)^ HPMECs. Therefore, we suspect the mechanism of AT-Lipoxin A4 protects the SAP-associated lung injury by affect TNF-*α*/TNF-R1 and downstream-related proteins.

High concentrations of proinflammatory cytokines cause the injury of alveolar endothelial cells [27]. In our study, systemic administration AT-Lipoxin A4 significantly attenuates the increase of serum TNF-*α* and IL-6 and alleviates pancreatic and lung tissue pathological damage. *In vitro*, we found that AT-Lipoxin A4 markedly suppressed the protein expression of VCAM-1 and E-selectin in TNF-*α*-exposed HPMECs and TNF-R2^(-/-)^ HPMECs. Taken together, we provided evidence that AT-Lipoxin A4 ameliorates SAP and SAP-associated lung injury. The outcomes of the study indicate that AT-Lipoxin A4 can break this waterfall-like vicious chain-reaction process, inhibit SIRS, and prevent this process so as to treat the critically ill.

MAPK is a serine/threonine protein kinase formed by a variety of isoenzymes, including extracellular signal-regulated kinase (ERK), P38, and JNK. It was shown that it is sufficient to prevent LPS-induced TNF-*α* generation if any of the three MAPK pathways is inhibited [28]. In addition, the ERK pathway is also involved in cell response caused by stress, bacterial products, and inflammatory mediators, indicating that the activation of the ERK pathway is closely related with various types of inflammation [29]. Studies have shown that p38 MAPK phosphorylation was increased in TNF-*α*-induced human pulmonary microvascular endothelial cells and the MAPK inhibitor can inhibit the expression of cytokines and adhesion molecules [30, 31]. MAPKs are a group of signaling molecules that appear to play an important role in inflammatory processes.

To further investigate the mechanism responsible for the anti-inflammatory effect of AT-Lipoxin A4, we examined the effect of AT-Lipoxin A4 on JNK MAPK and ERK MAPK phosphorylation in HPMECs and TNF-R2^(-/-)^ HPMECs. The results showed that AT-Lipoxin A4 inhibited TNF-*α*-induced JNK MAPK and ERK MAPK phosphorylation in HPMECs and TNF-R2^(-/-)^ HPMECs, which have shown that MAPK activation is attenuated in the presence of AT-Lipoxin A4. Considering our previous experimental results *in vitro* verifying the anti-inflammatory effect of AT-Lipoxin A4, these results suggest that the anti-inflammatory effect of AT-Lipoxin A4 may depend on the regulation of the JNK MAPK and ERK MAPK signaling pathway. Further, our previous results indicated that LXA4 inhibits p38 MAPK activation in HPMECs induced by TNF-*α* and that LXA4 alone did not significantly change it in HPMECs [20]. Therefore, the results have shown that MAPK activation is attenuated in the presence of AT-Lipoxin A4. Hence, it can be inferred that TNFR1 signaling is complex in activating MAPK signaling pathways, playing a pivotal role in the development of SAP-associated lung injury.

Taken together, our results clarified that AT-Lipoxin A4 can suppress the activation of TNF-*α*/TNF-R1 and downstream signals such as TRADD, TRAF2, and RIP, which results in the suppression of the NF-*κ*B pathway and MAPK pathway activity and in turn inhibits the generation of an inflammatory mediator. Downregulation of TNF-*α*/TNF-R1 signals by AT-Lipoxin A4 may be a significant mechanism of attenuation in SAP-associated lung injury.

## Figures and Tables

**Table 1:** The primers used in the real-time PCR.

Gene	Sense (5′-3′)	Antisense (5′-3′)
TNF-R1	CGGAAAGAAATGTTCCAGGT	CACTGGAAATGCGTCTCACT
TRADD	CTGCTGATGTTGCTACTGCTG	CAGGAAGTCCTCTTTCTGTAC
TRAF2	AAGGTCCCAATGATGCTCTC	TCTGGAAGGAGGACGAAGTT
RIP	TCATCATGGGTTTGGAGCTA	CAAGGAGATGTATGGCATGG
GAPDH	GTCTTCACCACCATGGAGAA	ATCCACAGTCTTCTGGGTGG

**Table 2:** Scores of pancreas and lung injuries (*n* = 8).

Groups	Pancreas (12 h)	Pancreas (24 h)	Lung (12 h)	Lung (24 h)
Sham	0.42 ± 0.09	0.46 ± 1.07	0.35 ± 0.06	0.34 ± 0.07
SAP	11.34 ± 1.23	13.38 ± 1.34	10.32 ± 1.32	11.43 ± 1.22
SAP+AT-Lipoxin A4	9.74 ± 1.08	10.23 ± 1.42	8.98 ± 1.13	9.98 ± 1.08
*P* value	<0.01	<0.01	<0.05	<0.05

SAP: severe acute pancreatitis; SAP+AT-Lipoxin A4: SAP+administration of AT-Lipoxin A4. *P* value: the statistical value of the SAP group vs. that of the SAP+AT-Lipoxin A4 group.

## Data Availability

The data used to support the findings are available from the corresponding author upon request.

## Abbreviations

ALI: Acute lung injury
HPMECs: Human pulmonary microvascular cells
H&E: Hematoxylin and eosin
IL-6: Interleukin-6
LXA4: Lipoxin A4
MODS: Multiple organ dysfunction syndrome
NF-*κ*B: Nuclear factor *κ*B
PMN: Polymorphonuclear neutrophils
RIP: Receptor-interacting protein
SAP: Severe acute pancreatitis
SIRS: Systemic inflammatory response syndrome
TBS: Tris-buffered saline
TNFR: Tumor necrosis factor receptor
TRADD: TNFR1-associated death domain protein
TRAF2: TNFR-associated factor 2
VCAM-1: Vascular cell adhesion molecule-1
ARDS: Acute respiratory distress syndrome.
